# Chicken Incubation Conditions: Role in Embryo Development, Physiology and Adaptation to the Post-Hatch Environment

**DOI:** 10.3389/fphys.2022.895854

**Published:** 2022-05-23

**Authors:** K. Tona, K. Voemesse, O. N’nanlé, O. E. Oke, Y. A. E. Kouame, A. Bilalissi, H. Meteyake, O. M. Oso

**Affiliations:** ^1^ Centre d’Excellence Régional sur les Sciences Aviaires, University of Lome, Lome, Togo; ^2^ Institut Togolais de Recherche Agronomique, Lome, Togo; ^3^ Department of Animal Physiology, Federal University of Agriculture, Abeokuta, Nigeria

**Keywords:** chicken, chick quality, physiology, metabolism, post-hatch growth, photo-incubation, circadian rhythm

## Abstract

The chicken hatching egg is a self-contained life-supporting system for the developing embryo. However, the post-hatch performance of birds depends on several factors, including the breeder management and age, egg storage conditions and duration before incubation, and the incubation conditions. Studies have determined the effect of incubation factors on chick post-hatch growth potential. Therefore, chick physical quality at hatch is receiving increasing attention. Indeed, although incubation temperature, humidity, turning and ventilation are widely investigated, the effects of several variables such as exposure of the embryo to high or low levels, time of exposure, the amplitude of variations and stage exposures on embryo development and post-hatch performance remain poorly understood. This review paper focuses on chick quality and post-hatch performance as affected by incubation conditions. Also, chick physical quality parameters are discussed in the context of the parameters for determining chick quality and the factors that may affect it. These include incubation factors such as relative humidity, temperature, turning requirements, ventilation, *in ovo* feeding and delay in feed access. All these factors affect chick embryo physiology and development trajectory and consequently the quality of the hatched chicks and post-hatch performance. The potential application of adapted incubation conditions for improvement of post-hatch performance up to slaughter age is also discussed. It is concluded that incubation conditions affect embryo parameters and consequently post-hatch growth differentially according to exposure time and stage of exposure. Therefore, classical physical conditions are required to improve hatchability, chick quality and post-hatch growth.

## 1 Introduction

The growth and metabolism of a 1-day-old chick are mostly determined by processes that occur during embryonic development. The major goal of farmers is to develop a chick with good hatchability, viability, and post-hatch performance. To reach this goal, it is vital to determine the sources of variable factors as well as the repercussions of these factors for optimal embryonic development and hatching outcomes. Physiological changes occur during embryonic development and the hatched day-old chick results in 21 days of development (Decuypere and Bruggeman, 2007). As a result, the endocrine system is absolutely necessary for appropriate embryonic development and hatching success.

The relationship between several physiological parameters such as corticosterone and thyroid hormones balances, heat production and metabolism, and gas exchange (O_2_, CO_2_) is crucial for the development of embryos and their survival under the incubation process (Decuypere et al., 1990; Tona et al., 2004). Furthermore, incubation conditions such as temperature, hypoxia (low oxygen), hyperoxia (high oxygen), and hypercapnia (high CO_2_) can alter these physiological parameters and influence embryonic development in various ways. This could have an impact on embryo general growth trajectory and, as a result, flock uniformity. In literature, the relationships between physiological parameters and incubation conditions with embryonic development in time are scarce, and a better understanding of these parameters that affect chick quality and post-hatch growth is highly desired. The link between the initial feeding and post-hatch chick performance is crucial. It is well known that denying 1-day-old chicks access to nutrition decreases post-hatch growth. It is widely known that the first feeding stimulates a variety of molecular and cellular targets, including enzymes and hormones, which affect general growth and a variety of physiological processes, including the yolk utilization, metabolic level, and gastrointestinal development (Decuypere and Bruggeman, 2007). As a result, the relationship between the initial feeding and post-hatch chick performance is particularly intriguing. *In-ovo* feeding was examined in-depth to understand how exogenous nutrients could affect embryonic growth and hatching. Additionally, photo-incubation, a process of stimulating developing embryos with light is also reviewed. During embryogenesis, the growth-promoting effect of photo-incubation has been reported and there are shreds of evidence that photo-incubation influences hatch events (Tong et al., 2018), post-hatch growth performance parameters (Zhang et al., 2016), fear responses (Archer et al., 2009), stress level and adaptability to novel post-hatch environment (Ozkan et al., 2012). The role of light in the physiological process of poultry ontogenesis is essential to synchronize knowledge and scientific findings. This review focuses on the effects of incubation conditions such as ventilation, light, temperature, relative humidity and *in ovo* feeding on embryo and post-hatch parameters.

## 2 Ventilation

Hypoxia (low O_2_), hyperoxia (high O_2_), and hypercapnia (high CO_2_) during incubation are known to have a positive impact on embryonic development, depending on the extent to which the embryo is exposed to these conditions and the stage of the embryo development. As a result, hatchery managers must understand the impacts of low O_2_, high O_2_, and high CO_2_ on embryo growth trajectory during incubation.

### 2.1 Effect of Hypoxia/Hyperoxia or Hypercapnia on Embryonic Development

#### 2.1.1 Effect of Hypoxia

It is widely known that the level of O_2_ in the atmosphere varies with altitude, implying that the risk of hypoxia exists. With higher altitudes, the oxygen rate declines, affecting incubation time and hatchability (Hassanzadeh et al., 2002). According to (Smith, 1933), incubation of eggs at high altitudes caused a delay in embryo growth. Zhang and Burggren (2012) reported that variations in normal chick embryo growth are dependent on the timing of hypoxia and on its severity, with lower O_2_ levels having a greater impact on growth and size.

Mild hypoxia (15 percent O_2_) is the most studied level of hypoxia because it poses a major hypoxic threat to the embryo without causing severe mortality. This interpretation is backed by Chan and Burggren (2005) findings show that embryo growth is reduced but smaller when exposed to 15% O_2_ hypoxia for 6 days (E1 to E6, E6 to E12, and E12 to E18) compared to controls. Furthermore, mild hypoxia (15 percent O_2_) during internal pipping reduced O_2_ intake and altered chick weight at hatching, but it had a minimal morphological influence on chicken embryos, whereas severe hypoxia (10 percent O_2_) compromised embryo viability (Szdzuy et al., 2008).

During the external pipping, responses to both levels of hypoxia increased (Menna and Mortola, 2003). Depending on the timing, short periods of hypoxia exposure throughout different time frames have varying impacts on embryo viability. During hypoxic incubation, Zhang and Burggren (2012) found that mortality was higher from E0 to E10 than from E11 to E18. This finding suggests that the first eleven days of incubation is the critical phase for the deleterious impact of hypoxia on embryonic development, whereas the last ten days is the crucial phase for the organs’ compensatory response to hypoxia.

#### 2.1.2 Effect of Hyperoxia

**FIGURE 1:**
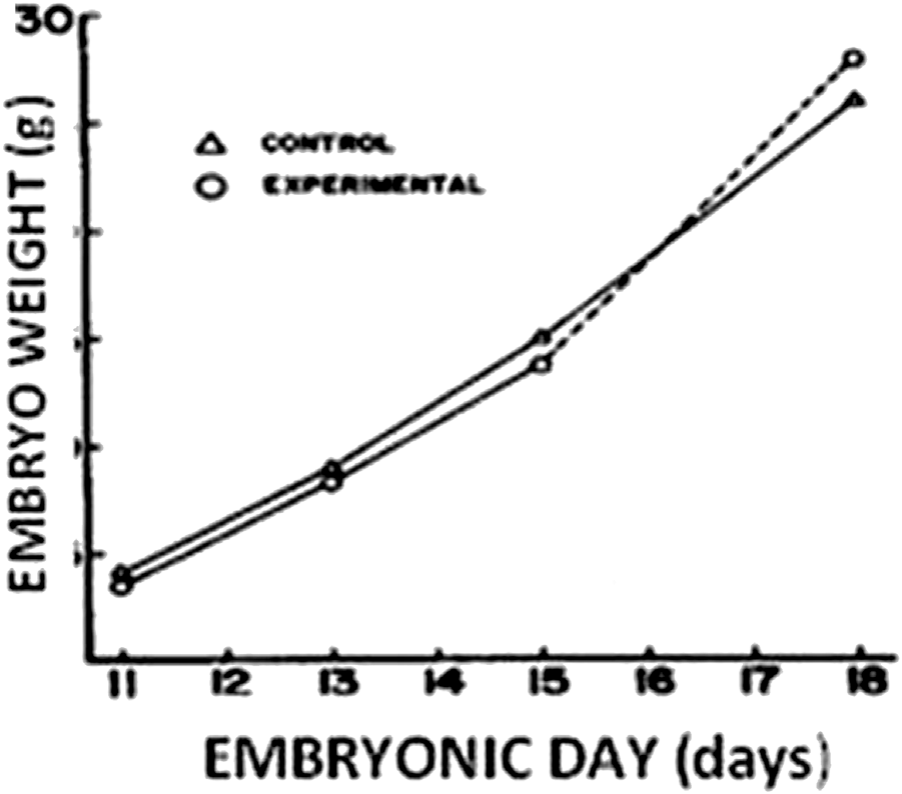
Chicken embryo weight (g) according to hyperoxia treatment (60% O_2_ on d 16–d 18) [adapted from Stock and Metcalfe (1987)].

The demand for oxygen surpasses the oxygen diffusion capacity of the egg-shell pore system and chorio-allantoic membrane in the last half of the incubation phase (Rahn et al., 1974), resulting in a decrease in O_2_ consumption (Prinzinger et al., 1995), and the development rate (Vleck et al., 1980). Internal pipping and the commencement of pulmonary respiration restore these modifications on day 19 (Prinzinger et al., 1995). As a result, the embryo outgrows the egg shell’s oxygen diffusion capability, and its growth may be restricted by the availability of oxygen during regular air incubation. As a result, raising O_2_ levels during the final stage of incubation can help the embryo grow faster. According to Stock and Metcalfe (1987), exposure to hyperoxia (60% O_2_) late in the incubation period (days 16–18) produces accelerated foetal development (Figure 1)**.** Furthermore, Van Golden et al. (1998) found that exposing the embryo to acute hyperoxia (60% O_2_ for 48 h) on days 10–11, 14–15, and 18–19 increases the embryo’s and all organs’ mass. However, it should be highlighted that previous studies on hyperoxia are outdated, and there are insufficient investigations on the impact of hyperoxia on embryo physiology and later performance. As a result, further investigations are needed in this area.

#### 2.1.3 Effect of Hypercapnia

Decuypere et al. (2006) found that chicken embryos become less susceptible to high incubator CO_2_ levels as they become older, similar to hypercapnia. Although hypercapnia during incubation was traditionally thought to be harmful to embryo development, recent research suggests that, depending on the timing of its occurrence, elevated incubator CO_2_ levels may be advantageous to the growing embryo (Onagbesan et al., 2007). Özlü et al. (2018) found that a higher CO_2_ concentration of 0.70% during the first three days of incubation lowered viable hatchability by 2 percent due to increased early embryonic mortality. This finding backs with Taylor and Kreutziger’s (1965, 1966) findings that indicated CO_2_ concentrations surpassing 1, 3, 6, 9, 8, and 7% between ED 0–4, 3–5, 9–12, 13–16, and 17–20 decreased hatchability. In a more recent study, El-Hanoun et al. (2019) found that duck breeder eggs incubated in a closed incubator with a carbon dioxide concentration of 1% at the end of the first 10 days of incubation had higher hatchability and embryonic growth. Everaert et al. (2007) discovered that exposing embryos to high CO_2_ (4%) during the second half of the incubation (d10–d18), had no influence on hatchability or hatch time but did increase embryonic weights. These reports show that the susceptibility of the chick embryo to CO_2_ changes with age, the same as it does with O_2_.

#### 2.1.4 Synergistic Effect of Hypoxia/Hyperoxia and Hypercapnia

It is proposed that CO_2_ levels of more than 6–7% have been demonstrated to drastically reduce O_2_ levels in the incubator, exacerbating the negative consequences of these high CO_2_ levels (Taylor et al., 1971). Restoring O_2_ levels to normoxic levels with high CO_2_ levels was observed to restore optimum hatchability in the later part of the incubation period, but restoration to hyperoxic levels induced an increase in hatchability relative to control incubations (Taylor and Kreutziger, 1969). This shows that high amounts of CO_2_ and O_2_ have a synergistic impact that may be beneficial to the growing embryo. These studies found that manipulating O_2_ or CO_2_ levels during incubation can influence the development of certain physiological regulating systems, producing alterations in the embryo’s development trajectory. As a result, one can wonder about the impact of hypoxia or hypercapnia on embryonic physiology during embryogenesis.

### 2.2 Effect of Hypoxia or Hypercapnia on Embryonic Physiology

**FIGURE 2:**
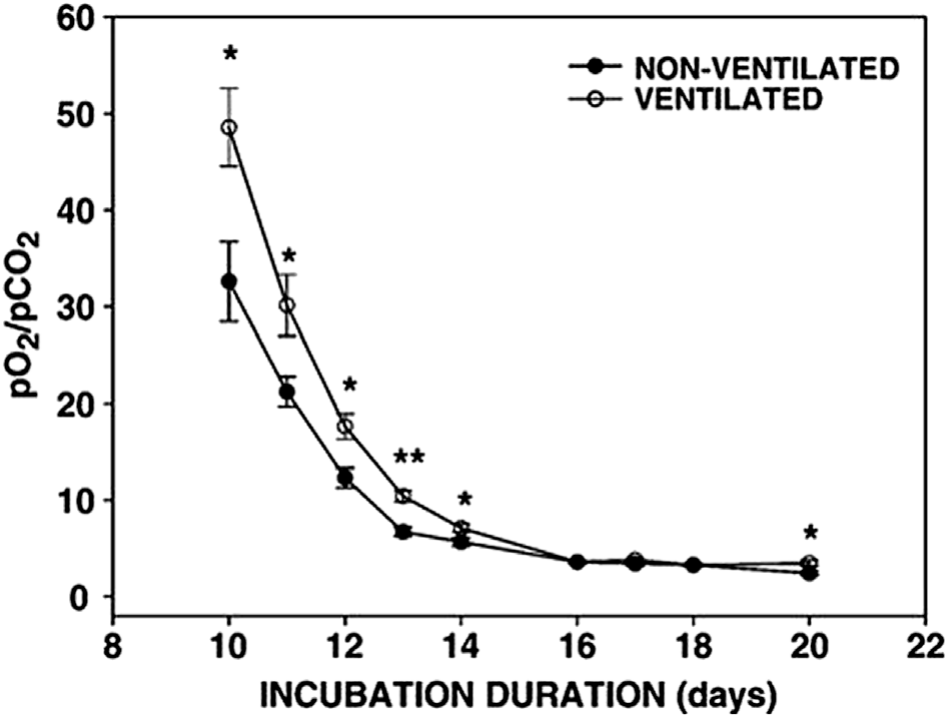
Changes in the ratio of the partial pressure of O_2_ and CO_2_ in the air cell of the developing egg. *p < 0.05; **p < 0.0001, De Smit et al. (2006).

Hypercapnia or hypoxia can cause changes in the physiology of embryos with respect to the control and timing of the hatching events The physiological changes can be induced in the pulmonary and circulatory system by chronic hypercapnia. This observation has resulted in the view that higher levels of CO_2_ can shorten the effects of hypoxic conditions on developing embryos. Increasing CO_2_ early or at the end of incubation acts as a hatching stimulus but also the hypoxic condition of high-altitude incubation also affects hatching events as well as hormonal levels. In fact, Hassanzadeh et al. (2004) showed that embryos incubated at high altitude had higher plasma triiodothyronine (T_3_), thyroxin (T_4_), and corticosterone levels and hatched earlier than those incubated at low altitude. El-Hanoun et al. (2019) reported that duck breeders’ eggs incubated under hypercapnic conditions hatched earlier than those incubated under normal conditions, and the hatch window was narrower. The authors demonstrated that this phenomenon is strongly related to increased levels of corticosterone, T_3_ and T_4_ as a result of increased pCO_2_ (De Smit et al., 2006) in the air cell at internal pipping in hypercapnic condition (Figure 2). Thus the positive effects of hypercapnic incubation suggest an increase of T_3_ and air cell pCO_2_ resulting in the early hatch and enhanced hatchability.

The study of Tona et al. (2004) on non-ventilation during early incubation in combination with dexamethasone administration at two stages of development (d16 or d18) in embryos has elucidated the importance of timing in manipulating the hypothalamic-pituitary-adrenal (HPA) axis. Indeed, the authors reported that dexamethasone injected on day 18 raised the plasma T_3_ levels (Table 1) at internal pipping (IP) and advanced hatching and reduced the hatching process. However, injection on day 16 had no effect on the plasma T_3_ levels at IP. Also, dexamethasone injection on day 16 resulted in a rebound effect on the functioning of the HPA axis in early postnatal life, which was not observed in chickens injected on day 18. This disturbance in HPA axis establishment may cause an increased functioning and has been reviewed earlier (Decuypere and Michels, 1992).

**TABLE 1:** Hormone levels (ng/ml) at day 18 of incubation according to incubation treatments (IT) and dexamethasone administration at day 16 (Dex 16) (*n* = 24) of incubation or control eggs (Cont) (*n* = 40).

IT	Groups	T_3_	T_4_	Ratio,T_3_/T_4_	Corticosterone
NV	Count	0.11 ± 0.01^c^	3.94 ± 0.26^b^	0.03 ± 0.01^c^	8.19 ± 1.04^a^
	Dex 16	0.32 ± 0.04^a^	5.47 ± 0.52^a^	0.08 ± 0.02^a^	2.91 ± 0.99^b^
V	Count	0.11 ± 0.01^c^	3.52 ± 0.57^b^	0.05 ± 0.02^b^	7.72 ± 1.15^a^
	Dex 16	0.23 ± 0.04^b^	5.51 ± 0.90^a^	0.05 ± 0.01^b^	4.61 ± 1.44^b^

Within columns, data sharing no common letters (a–c) are different (*p* < 0.05). Adapted from Tona et al. (2004).

Moreover, exposure of embryos to low O_2_ or higher CO_2_ resulted in significantly higher haematological parameters (Hb, PCV %, and RBC counts). Increased Hb under hypercapnia or hypoxia conditions is known to raise the oxygen-carrying capacity of the blood and represent an adaptive physiological response. The findings of Mortola (2004) indicated a stimulatory role of CO_2_ on the chemoreceptors that enhance breathing efficiency and that hyperoxia at this period decreased the effect of hypercapnia. Hence, hypercapnia can achieve a similar effect as hypoxia on lung function during internal and external pipping and hatching.

These findings indicate that embryos adapted to hypoxic or hypercapnic conditions by enhancing angiogenesis processes, which subsequently increases their blood oxygen-carrying capacity, which positively affects their growth development and maturation. Such alterations may induce permanent phenotypic changes in the embryo, which may have a long term epigenetic effect on post-hatch performance.

### 2.3 Effect of Hypoxia or Hypercapnia on Embryonic Post-Hatch Growth

#### 2.3.1 Effect of Hypoxia

The use of moderate to high hypoxia is beneficial to chicken embryos during incubation as it supports the cardiovascular development chorioallantoic membrane, leading to an enhanced oxygen-carrying capacity and resulting in developmental plasticity which can influence the tolerance and performance of chicks to stressful conditions during their post-hatch growth.

**FIGURE 3:**
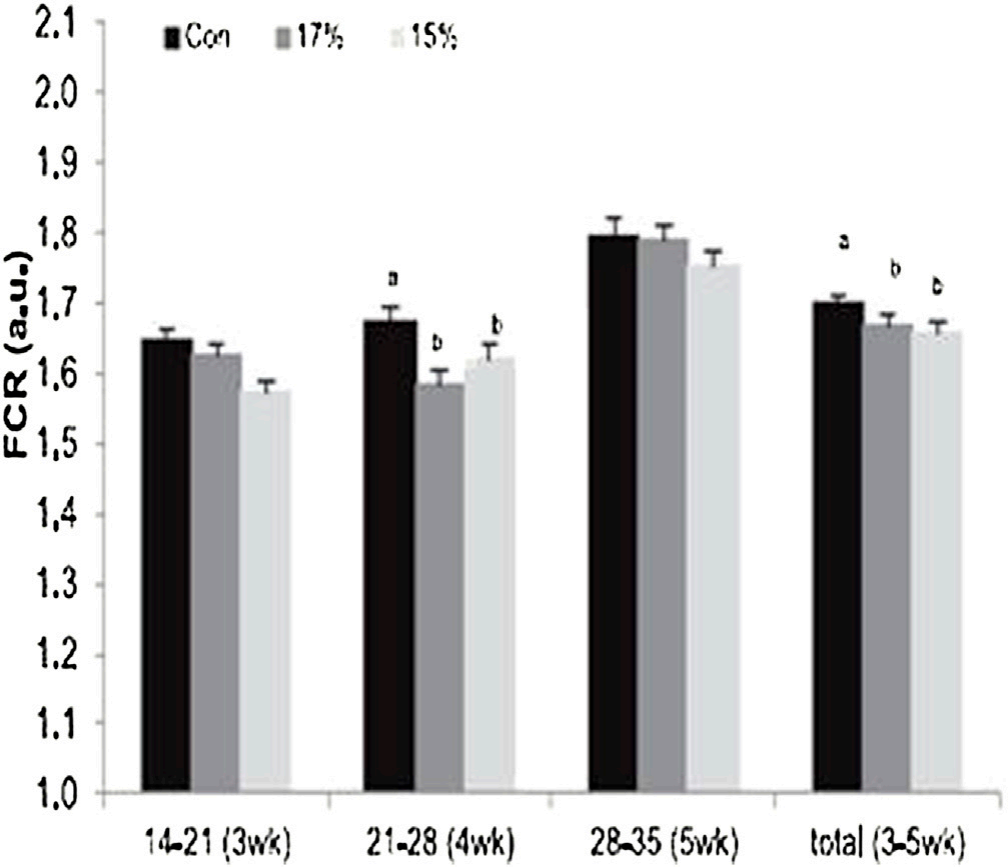
Effect of hypoxia (17 or 15% O_2_) on feed conversion ratio [adapted from Druyan et al. (2018)]. FCR, feed conversion ratio.

Chronic hypoxic conditions retarded growth rate in the initial phase in the first 14 days post-hatch but there was no difference in the body weights in the later phase of growth (Hassanzadeh et al., 2004). Also, the findings of Huang et al. (2017) showed that chronic hypoxia condition adversely affected survivability, feed conversion ratio and growth in broiler chickens. On the contrary, the findings of Druyan et al. (2018) showed that hypoxic conditions did not alter the juvenile growth performance of broiler chickens using a hypoxic condition of 15 or 17% O_2_ during a short period of embryonic development. The authors reported that hypoxic conditions improved the body weights of the birds at the market age. At weeks 3 and 4, the treated birds had higher growth and a better feed conversion ratio (FCR) (Figure 3).

#### 2.3.2 Effect of Hypercapnia

Relative to hypercapnia, El-Hanoun et al. (2019) reported higher body weights, better feed intake and FCR in the first 6 weeks of age of ducks incubated under hypercapnic conditions induced by non-ventilation during the first 10 days of incubation. These findings were in agreement with the results reported by Fares et al. (2012) and De Smit et al. (2006). The findings of De Smit et al. (2006) showed that the differences in body weight were due to the higher growth speed of chicks from nonventilated incubated eggs in the first week post-hatch compared to those from the ventilated incubation. They maintained higher bodyweight during the entire post-hatch growing period which suggests a long term epigenetic effect of non-ventilation. Based on this epigenetic effect, in literature, it has been hypothesized that the negative impact of long-term storage can be compensated by increasing CO_2_ levels in the incubator during incubation.

Developmental changes induced by increasing dioxide carbon or oxygen level during embryonic development may play a role in post-hatch performance, affecting growth and metabolism (Decuypere, 2002). Although later prenatal hypoxia, as well as hypercapnia, may be beneficial for a lower incidence of ascites during the growing period of broilers, early hypercapnia as induced by non-ventilation during the first 10 days of incubation may result in increased sensitivity for ascites-inducing factors (De Smit et al., 2008). This shows that the timing of the treatment influences the lasting epigenetics of this condition.

## 3 Light

Photo-incubation is a complex phenomenon whose outcome can be determined by a certain number of factors which can be categorized as the bulb type, wavelength and correlated colour temperature (CCT), time of initiation of photo-incubation, light duration and light intensity. During embryogenesis, the growth-promoting effect of photo-incubation has been reported and there are pieces of evidence that light-dependent factors influence hatch events (Tong et al., 2018), post-hatch growth performance parameters (Zhang et al., 2016), fear responses (Archer et al., 2009), stress level and adaptability to novel post-hatch environment (Ozkan et al., 2012). It is known that the ability of birds to adapt to the prevailing post-hatch environment has been linked to physiological roles played by biological rhythm established during embryogenesis (Ozkan et al., 2012). Therefore, reviewing the importance of light dependent factors on developing embryo and their effect on post-hatch growth is essential to synchronize knowledge and scientific findings.

### 3.1 Effect of Light Characteristics on Embryonic Development and Physiology

#### 3.1.1 Effect of Bulb Type on Embryonic Development and Physiology

Bulb type, which serves as a light source, is a crucial factor that can potentially enhance or disrupt the process of photo-incubation. For instance, the problem of secondary heating associated with incandescent (ICD) due to its high heat emitting capability could engage the mechanism of thermal physiology in the process. The usage of ICD is highly discouraged if the bulb is not intended to be used as a primary source of heat in the incubator. Studies have demonstrated that other bulb types such as fluorescent and LED posed lower (Rozenboim et al., 2004) or no secondary heating effect during incubation (Zhang et al., 2016). A comparative study on light sources revealed that fluorescent light enhances the embryonic weight of quail over ICD. Incandescent light decreased hatch weight and hatchability but increased early and late embryonic mortality in contrast to fluorescent (Hanafy and Hegab, 2019). It was also reported that small-sized eggs develop faster under ICD while the rate of development under fluorescent was not influenced by egg size (Hanafy and Hegab, 2019).

#### 3.1.2 Effect of Light Duration on Embryonic Development and Physiology

Duration of light exposure or photoperiod is an essential photo-incubation parameter. Non-continuous or intermittent lighting (12 h of light) seems beneficial over other photoperiods. Studies have shown a reduction in embryonic mortality (Riaz et al., 2021) and an increase in melatonin hormone under intermittent lighting on day 19 of incubation (Archer and Mench, 2014a) compared to continuous or dark incubation. Continuous photo-regimen (23 or 24 of light) has been reported to elevate eggshell temperature (Rozenboim et al., 2004) and a destructive effect of the regimen has also been reported on avian eyes (Archer et al., 2009). Interestingly, Raiz et al. (2021) reported a shorter hatch window and improved hatchability under both continuous and intermittent in contrast to dark incubation. In contrast, Archer and Mench (2014b) reported no impact of lighting duration on hatchability relative to dark incubation. A factor confounding these studies might be the differences in intensities used by the authors.

#### 3.1.3 Effect of Light Intensity on Embryonic Development and Physiology

An existing study on light intensity showed that the use of the fluorescent green light at 900–1,380 lux and 1,430–2,080 lux, had no significant influence on the embryonic weight and hatch weight of broiler eggs (Shafey et al., 2005). Embryonic response to wavelength differs. Rozenboim et al. (2004) found an increase in embryonic weight of chicken eggs stimulated under green LED (at 0.1 W/m2 intensity, phot-incubated from day 5–21) relative to dark incubated eggs. Zhang et al. (2016) noted that light colours (White LED, green LED-560 nm supplied at 30 lux) had no influence on hatchability or hatching weight as compared to dark incubation. Tong et al. (2015) and Wang et al. (2020) reported that green LED (522 nm, 520–525 nm) shortens hatch time over dark incubation. In contrast, no effect of incubation condition (green LED or darkness) was recorded on hatchability (Zhang et al., 2016) hatching weight (Tong et al., 2015; Zhang et al., 2016) and chick quality (Tong et al., 2015). Sabuncuoglu et al. (2018) reported no differences in hatch weight, hatch time, and hatchability of quail hatching eggs incubated in the dark, blue (480 nm) or green (560 nm) LED. Green LED (565 nm at 15 lux) has been reported to increase growth hormone (GH) and insulin-like growth factor (IGF-I) during embryonic development (Zhang et al., 2014) while the prehatch level of T_3_ and T_4_ remains unchanged. Differences in intensities due to light distribution might influence embryonic response on an individual level. Thus, it is essential for researchers to report light intensities based on average measurements recorded at the egg level. It is also imperative for researchers to report light intensities in gallilux or chicken lux rather than lux as poultry birds and humans perceive light differently (Oso et al., 2022a,b). This is important, especially when photo-incubation extends till hatch or birds are light stimulated post-hatch.

#### 3.1.4 Effect of Onset of Photo-Incubation/Total Duration of Photo-Incubation on Embryonic Development and Physiology

The onset of photo-incubation/total duration of photo-incubation is another factor due for consideration during photo-stimulation. Archer and Mench (2014b) demonstrated that initiating photo-incubation either from day 1 or day 7 or day 14 till hatch had no impact on hatchability. Similarly, Hannah et al. (2020) proved that initiating photo incubation on days 0, 9, and 17 till hatch had no influence on hatchability and embryonic mortality. Exposing eggs to light from day 1–18 or day 1–21 has been demonstrated to have no effect on embryonic weight and hatch weight of chicks in comparison to dark incubation (Archer, 2015). Scientific knowledge is limited on the effect of the varying onset of photo-incubation on physiological indices, especially hormones during embryonic development and at hatch.

#### 3.1.5 Other Factors That can Influence the Photo-Incubation Process

Photo-incubation is a complex phenomenon whose outcome cannot only be determined by light-dependent factors but also by other factors known as egg dependent factors. Egg dependent factors include egg internal qualities, shell characteristics, specie, breed and strain, storage duration of the egg before incubation, age of parent stock, size of an egg, and stage of embryonic growth/development at the time of photo-initiation (Shafey et al., 2005; Hannah et al., 2020). A crucial egg-dependent factor is the shell qualities. Eggshell thickness and shell pigmentation are capable of changing an embryo’s perception of light, thus influencing its response to photo-stimulation (Shafey et al., 2005). Darker eggshells change the wavelength perceived by the embryo in contrast to lightly-coloured eggs (Hannah et al., 2020). The differences in the outcome of photo-incubation based on the factors above suggest that the mechanism of photo-incubation may slightly differ under varying conditions; however, the site(s) of photo-stimulation during embryogenesis remains the same.

### 3.2 Effect of Photo-Incubation Light Factors on Post-Hatch Growth Development and Physiology

#### 3.2.1 Effect of Bulb Type on Post-Hatch Growth Development and Physiology

The effect of bulb type used during photo-incubation on post-hatch growth and physiology of many poultry species remains un-elucidated. More often than not, photo-incubated chicks are reared under lights that differ from the incubation light source. In Japanese quails, Hanafy and Hegab (2019) reported no significant influence of ICD and fluorescent bulb type used during incubation on post-hatch body weight, weight gain and FCR at 6 weeks; however, birds belonging to the fluorescent group had significantly higher feed intake group compared to those in ICD group. The bulb type used during post-hatch was not stated by the author, although the author noted that the post-hatch lighting condition was the same. It is not known if maintaining the incubation light source during post-hatch could influence the growth and physiology of photo-incubated birds differently, thus, a comparative study is necessary in this regard.

#### 3.2.2 Effect of Light Duration on Post-Hatch Growth Development and Physiology

The effects of incubation photo-period on post-hatch growth performance reported in the literature are contradicting. Both intermittent and continuous lighting were reported to have no influence on feed intake, weight gain (Archer and Mench, 2013; Riaz et al., 2021) and feed conversion ratio (FCR) (Archer and Mench, 2013) in contrast to dark incubation. On the contrary, both continuous and intermittent duration had similarly been demonstrated to reduce post-hatch feed intake over dark incubation. Interestingly, continuous lighting during incubation had been shown to significantly reduce post-hatch weight gain in contrast to dark incubation and non-continuous (Yameen et al., 2020). Riaz et al. (2021) highlighted that FCR was significantly better under intermittent photo-incubation over continuous or dark incubation. A likely interactive effect between pre-hatch and post-hatch photo-period and other lighting conditions might have confounded the results of these studies and this appears to be another promising area of research. The effect of incubation light duration on melatonin appears to wane with time or fades out due to prevailing post-hatch lighting duration. Archer and Mench (2014a) demonstrated that changes in pre-natal melatonin level were not sustained till 5 weeks post-hatch as no significant difference was observed in the melatonin level between the continuous, non-continuous or dark incubation groups. It is indistinct if post-hatch photoperiod over-rides the effects of pre-hatch photoperiod, but certainly, the circadian rhythm established during the last phase of photo-incubation under an established non-continuous regimen is beneficial to post growth and development either directly or indirectly. Before exposure of birds to a stressful situation (crating exercise), Archer and Mench (2013) recorded a similar level of corticosterone in chickens exposed to continuous lighting, intermittent and near intermittent (6 h of light). After exposure, a lower level of corticosterone was reported in birds belonging to the intermittent group when compared to other groups. This suggests that the incubation photo-period has a vital role to play in post-hatch stress management. The Interactive effect of pre-hatch photo-period and prevailing post-hatch photoperiod on growth and physiological indicators in poultry birds has not been explored and this might be a promising area of research requiring considerable attention.

#### 3.2.3 Effect of Light Intensity on Post-Hatch Growth Development and Physiology

The effect of pre-hatch light intensity on post-hatch growth, physiology and adaptation of poultry birds is not established in the literature and a probable interaction between pre-hatch light intensity and post-hatch intensity has not been researched. Different light intensities are required by different poultry birds at different stages of post-natal growth and this might extend to photo-incubation. Establishing intensity specifications required by each poultry species for a maximum photo-incubation outcome would further broaden photo-related scientific horizons.

It appears that the impact of photo-incubation wavelength on post-hatch growth performance varies between species, breeds or strains. Wang et al. (2020), who experimented with layer breeder eggs reported an increase in 8–12 weeks body weight of Rhode Island Red photo-incubated with green LED in contrast to dark incubated, but these differences disappeared from 14 weeks, whereas body weights of other strains (Columbia Rock, White Leghorn, Barred rock) remained unchanged throughout regardless of the incubation treatment. Sabuncuoglu et al. (2018) demonstrated that pre-hatch light colour (blue, green) does not influence the post-hatch bodyweight of quails throughout the rearing period. Rozenboim et al. (2003) showed that female turkeys earlier photo-incubated under green LED had higher body weight from day 28 till day 59 compared to those incubated in the dark. In another experiment published in the same article, the author recorded no difference in post-hatch body weight between male turkeys photo-incubated under green LED, white mini-ICD, or dark incubated. These results suggest that sex might play an important role in post-hatch growth response to incubation light colour. Zhang et al. (2016) highlighted that at 30 lux, green LED photo-incubation enhanced the weight gain of broiler chicks at 6 days old over dark incubation though the result was similar to those obtained in the white LED group. Furthermore, the author noted that feed intake and FCR were not influenced by the photo-incubation condition (Zhang et al., 2016). Prevailing post-hatch light colour might overwrite the effect of pre-hatch light colour. Rozenboim et al. (2004) found an increase in body weight of broilers photo-stimulated under green LED and reared under the green light (green-green) compared to those incubated in the dark and reared under white LED (dark-white). Although, the author demonstrated that green-green birds and green-white birds (photo-incubating with green and rearing under white light) had similar body weights. The significant differences emanating when comparing green-green and dark-white suggest that rearing birds under their incubation light colour might be more beneficial. Studies on hormones showed that green light enhances GH and IGF-I during post-hatch life (Zhang et al., 2014) compared to dark incubation and at slaughter age, but no differences within treatment groups were found in the T3 and T4 levels post-hatch. Archer (2017) reported a lower level of corticosterone and serotonin hormones in birds incubated under green, red and white LED compared to dark incubated birds.

#### 3.2.4 Effect Onset of Photo-Incubation on Post-Hatch Growth Development and Physiology

The onset of photo-incubation seems to have a more pronounced effect on hormones rather than growth performance. At post-hatch, photo-stimulation initiated from day 1 or day 7 or day 14 till hatch does not influence feed intake, weight gain and FCR (Archer and Mench, 2014b). Similarly, exposing eggs to light from day 1–18 or day 1–21 has no impact on weight gain and FCR at slaughter age. Before and after exposure to a stressful situation, Archer and Mench (2014a) recorded a significant reduction in corticosterone levels of birds exposed to light from day 1–21 and day 7–21 when compared to those incubated in the dark. Corticosterone level was found to be similar within the photo-incubated treatments but significantly lower than in the dark incubated groups (Archer, 2015). Dishon et al. (2021) demonstrated that exposing eggs to green LED from day 18 till hatch and photo-incubating them from day 1 till hatch significantly improve body weight at slaughter age. Also, at 5 days post-hatch, secretion of GH was higher in birds photo-incubated from day 18–20 in contrast to those incubated and hatched in the dark.

## 4 Incubation Temperature and Relative Humidity

One of the crucial determinants of the development of chickens’ embryos and hatchability is incubation temperatures (Decuypere and Michels, 1992). According to Molenaar et al. (2011), a constant temperature of 37.8°C during incubation allows for optimal embryo development, the best hatchability and the highest chick quality. It has been shown that a 1°C change from the optimum temperature can have a significant effect on hatchability (French, 1994). Additionally, the period of temperature change and its intensity and the age of the embryos during incubation will determine its impact on the developing embryos (French, 2002).

### 4.1 Effect of Low and High Incubation Temperature on Embryo Development and Hatching Performances

Joseph et al. (2006) highlighted that a continuous low eggshell temperature of 36.6°C during the first 10 days of incubation reduced embryonic weight, hatchability and chick quality. Nideou et al. (2019) observed an increase in albumen utilization and growth of Isa brown embryos subjected to elevated temperature (38.5°C) during the first 10 days of incubation while the incubation time was shortened. According to Maatjens et al. (2017), a negative effect of an eggshell temperature of 38.9°C was observed from E15 onward and emphasize that an eggshell temperature of 35.6 and 36.7°C from E15 onward might be beneficial for chick embryo physiology. However, Yildirim and Yetisir (2004) studied the effect of different hatcher temperatures (36.1; 37.2; 38.3, and 39.4°C from 17 days of incubation until hatch with relative humidity at around 75% in all groups) on hatching traits. They found that the control group (37.2°C) had a better hatchability compared to the low-temperature group (36.1°C) which had low metabolic activity and then a high late embryo mortality rate. Wilson (1991) reported that in the last third of the incubation phase, a decrease in the temperature of incubation did not have a significant effect on hatchability but elongated incubation duration and decreased water loss. According to Tzschentke and Hall (2009), a temperature of 36.2°C from day 18–21 of incubation improved hatchability. Concerning the effect of a high temperature in the hatcher; Yildirim and Yetisir (2004) found that a temperature of 38.3°C in the hatcher allows for a hatchability similar to the control group (37.2), but a very high temperature (39.4°C) negatively affect hatchability by increasing late embryo mortality. Their results are not similar to those of Joseph et al. (2006) who found a better hatchability with eggs subjected to a higher hatcher temperature (39.5°C) compared to the control group (37.8°C).

### 4.2 Effect of Low and High Incubation Temperature on Post-Hatch Performances

Joseph et al. (2006) found that a continuous low eggshell temperature of 36.6°C during the first 10 days of incubation reduced live weight at 6 weeks of age and carcass yields. But increasing the incubation temperature (38.5°C) during the first 10 days of incubation did not impact the T_3_ concentration of the hatched chicks and their post-hatch performance. Maatjens et al. (2016) showed that a high eggshell temperature (38.9°C) applied from day 15 of incubation on Ross eggs negatively impacted chick’s growth and FCR during the first week of rearing, while this post-hatch performance was improved compared to control batches when eggs were subjected to a temperature of 36.7°C from day 15 of incubation. According to İpek and Sözcü (2015), the carcass weight and yield are negatively affected by higher hatcher temperature but slaughter weight at higher hatcher temperature was similar to the control group. Joseph et al. (2006) stated in their study that high eggshell temperature in the hatcher reduced bodyweight and one-week weight gain. However, by three weeks of age, there was no difference in body weight between chicks in high eggshell temperature and control eggshell temperature treatments.

### 4.3 Effect of Duration of Thermal Treatment During Incubation on Embryo Development and Chickens’ Post-Hatch Performance

Increasing incubation temperature at 39.5°C during 24 h/day from E7 to E16 reduced hatchability by 25% and negatively affected the quality and the bodyweight of the hatched chicks while a thermal treatment at 65% during 12 h/day from E7 to E16 did not affect hatchability and bodyweight of cobbs hatched chicks (Piestun et al., 2008a). Tzschentke and Hall (2009) revealed that neither short-term (38.2°C–38.4°C, 2 h daily) nor chronic (38.2°C–38.4°C, 24 h daily) increase in incubation temperature in the hatcher (during the last four days of incubation) adversely affected hatchability and chick quality in broiler chickens. The body temperature was significantly reduced in broiler chicks subjected to thermal treatment (12 and 24 h/day) compared to the control group, but those subjected to a thermal treatment during 24 h/day had a body temperature lower than those of 12 h/day (Piestun et al., 2008a). This aligns with the reduced plasma triiodothyronine and thyroxine of the birds of 24 and 12 H groups and the plasma corticosterone increased with time.

### 4.4 Effect of Duration of Thermal Treatment During Incubation on Post-Hatch Performance and Physiology

Increasing incubation temperature at 39.5°C during 12 h/day from E7 to E16 improved the feed conversion ratio of broilers compared to the control (Piestun et al., 2013; Meteyake et al., 2020) without affecting broilers' growth compared to 24 h/day group which had lower body weights (Piestun et al., 2008b). According to Tzschentke and Hall (2009), the FCR of short-term warm stimulated broilers (38.2–38.4°C, 2 h per day, from d17 onward) was significantly lower than in broilers of the control (37.2–37.4°C) and chronic warm (38.2–38.4°C, 24 h per day, from d17 onward) incubated groups. The daily feed intake and weight gain were significantly lower in the short-term warm stimulated ducks than those of the control group in the first three weeks while short-term cold stimulation improved feed conversion ratio during the whole growing period exclusively in male ducks (Halle et al., 2012). According to Sgavioli et al. (2016), incubating eggs at 39°C compromises the body and heart development of layer chicks and reduces the availability of blood ionized calcium for bone mineralization during embryo development. Morita et al. (2016) concluded in their study that changes in chicken stickiness and vascularity as well as changes in thyroid and growth hormone levels are the results of embryonic strategies to cope with higher or lower than normal incubation temperatures. Overall, Madkour et al. (2021) claimed that thermal acclimation at the postnatal stage or throughout the embryonic stages has been considered as a novel promising strategy to mitigate the detrimental effects of heat stress in poultry. These authors suggested that, for large-scale application, this strategy needs further investigation to determine the suitable temperature and poultry age.

### 4.5 Relative Humidity

There is a loss in egg weight during incubation due to the evaporation of water (Rahn et al., 1977). This is crucial to make available ample air needed for the lung ventilation of the embryos sequel to internal pipping and ultimately hatching (Ar and Rahn, 1980). The best hatchability has been attained with 12–14% water loss at embryonic day 18 (Ar and Rahn, 1980). The relative humidity in the incubator can be manipulated to control the water evaporation of the eggs during incubation (Buhr, 1995). Lower or higher relative humidity could have variable effects on hatching and post-hatch performances.

Van der Pol et al. (2013), investigated the effect of low or high relative humidity and showed showed that incubating eggs at a higher or lower relative humidity negatively influenced hatching performances. They found that at the same incubation temperature (37.8°C), the reduction of relative humidity increased egg weight loss at E18 and reduced hatchability by increasing late embryo mortality. But it did not affect the chick’s weight, quality and hatching time. They found that incubating eggs at a low RH compared with a high RH and maintaining the EST at 37.8°C decreased the hatch of fertile eggs. Bruzual et al. (2000) reported that relative humidity of less than 63% during incubation decreased chick weight. Reducing relative humidity during incubation had little effect on post-hatch performance according to Van der Pol et al. (2013). El-Hanoun et al. (2012), investigating the effect of incubation humidity and flock age on hatchability traits and post-hatch growth in Pekin ducks found that the optimal relative humidity depends on breeders’ flock age.

### 4.6 Interaction Between Temperature Incubation and Relative Humidity

It is well known that a temperature of 37.5–37.8°C and relative humidity of 55–60% are optimal environmental conditions for efficient embryo development, and the best hatching and post-hatch performances. Any change of one of these two factors without changing the second accordingly could have variable effects on incubation results. (Boleli and Queiroz, 2012) found an interaction of these two incubation parameters on hatchability. They also found a moderate negative correlation between hatchability and temperature (r = −0.41), hatchability and egg weight loss (r = −0.31) and a positive correlation between hatchability and relative humidity (r = 0.47). The effect of the interaction of temperature and relative humidity was also significant on incubation duration and chicks’ body weight.

## 5 Egg Turning During Incubation

### 5.1 Effect on Physiology and Embryo Development

Egg turning involves four major factors that can be taken into account; the position of eggs, angle of turning, frequency (times/day) and stage of incubation in whom this turning occurs. These different factors of turning diversely influence the physiology of the embryo, incubation parameters and post-hatch performances.

The lack of turning during incubation has been reviewed (Lundy, 1969; Baggott et al., 2002). A complete absence of turning during the first but not the second week of chicken eggs incubation leads to an increase in mal-positioned embryos and mortality (Elibol and Brake, 2004). The results of New (1957) showed that days 3 to days 7 of incubation were critical and failure to turn eggs during this critical period leads to a decrease in hatchability and rates of embryonic growth (Deeming, 1989). Moreover, the lack of turning of chicken eggs between days 12 and days 19 of incubation (last stages of embryonic development) leads to less embryonic growth as a result of impaired O_2_ consumption through the chorioallantoic gas exchanger (Pearson et al., 1996). Tona et al. (2003a) noted that turning of eggs until 12, 15, and 18 d of incubation did not affect the levels of plasma corticosterone in the developing embryo or newly hatched chicks. The author inferred that corticosterone might not be involved in the mechanism by which turning affect hatching and production parameters. However, they observed that turning beyond 15 days increased pCO_2_ in air cells (hypercapnia) and plasma levels of T_3_ and T_4_ at the internal pipping. In contrast to Tona et al. (2003b) who showed a correlation between this increase in metabolism, hatching time and hatchability, these authors observed that these parameters remain similar between eggs turned until 12 days and 18 days, suggesting the involvement of other intrinsic factors.

Egg turning at 90° and 45° on either side of the vertical using as a standard practice in the industry was linked to considerable research during the 1930s–1950s (Baggott et al., 2002). Lesser turning angles increase mal-positioned and embryo mortality in domestic fowl (Funk and Forward, 1953; Elibol and Brake, 2006; Cutchin et al., 2009). Schalkwyk et al. (2000) noticed that hatchability amounted to 1·83% for an increase of 1° (*R* ^2^ = 0·96) when ostrich eggs were rotated hourly through angles ranging from 60° to 90°. In a recent study, Guo et al. (2021) recorded a shortened incubation duration and improvement in hatchability and goslings’ quality when eggs were turned at a wider angle (60° compared with 50°). They also observed an increase in late embryos and goslings’ weight correlate with a significant upregulation of genes in the somatotropic axis (GHRH, GH, and IGF-1 mRNA expression) and muscular development (pax7, MyoD, MYF5, and MRF4 mRNA expression). The authors concluded that wider angle turning made full use of the albumen content in goose eggs and recommend adjusting the angle of turning to the ratio of albumen in eggs according to avian species as previously stated by other researchers (Baggott et al., 2002; Elibol and Brake, 2006; Deeming, 2009).

Static incubation impairs the expansion of the *area vasculosa* during the critical period of sub-embryonic fluid production in the domestic fowl (Baggott et al., 2002). This absence of egg turning delayed the formation of extra-embryonic fluids and reduced rates of embryonic growth later in embryonic development (Deeming, 1989). Turning once an hour is commonly used in the industry concerning fowl eggs. The increase of this frequency up to 96 times daily does not significantly improve incubation results (Freeman and Vince, 1974). By contrast, lower frequencies decrease these results. Indeed, Oliveira et al. (2020) showed a decrease in hatchability due to a gradual increase percentage of early and late mortality with less turning frequency (Table 2). Elsewhere, a study conducted by Elibol and Brake (2006) showed that increasing turning frequency is a good way to reduce mal-positioned embryos associated with less turning angle during incubation. These data suggest possible interactions between the different factors of turning that are not well explored.

**TABLE 2:** Fertility, hatchability of fertile eggs, and embryonic mortality according to the turning frequency (Oliveira et al., 2020).

Turning frequency (time/D)	Fertility^2^ (%)	IIatchability of set eggs^3^ (%)	IIatchability of fertile eggs^4^ (%)	Early dead (%)	Mid dead (%)	Late dead (%)
24	93.00 ± 3.393^a^	85.34 ± 2.30^a^	91.84 ± 2.73^a^	2.84 ± 1.89^b,c^	1.41 ± 0.87^a^	3.57 ± 1.39^b^
12	91.33 ± 1.96	78.34 ± 2.30^b^	85.77 ± 3.05^b^	6.22 ± 1.99^b^	2.19 ± 0.73	5.46 ± 0.69^a,b^
6	90.67 ± 2.53	70.33 ± 5.3.31^c^	77.75 ± 3.89^c^	12.45 ± 2.05^a,b^	2.59 ± 0.83	7.37 ± 3.37^a,b^
3	91.56 ± 4.27	67.55 ± 5.5.82^c^	73.75 ± 3.89^c^	14.31 ± 1.82^a^	2.92 ± 0.64	8.05 ± 1.24^a^
*p* value	0.13	<0.0001^5^	<0.0001^5^	<0.0001^5^	0.11	0.02^5^
CV (%)	3.89	4.04	3.74	22.12	38.69	31.02
R^2^ adjust	0.23	0.81	0.84	0.81	0.25	0.38

Within columns, data sharing no common letters (a–c) are different (*p* < 0.05).

Moraes et al. (2018) incubated Japanese quail eggs in different positions (vertical position with the small end up, vertical position with the small end down, horizontal position) without turning and found that hatchability is best when eggs were set with the small end down although the outcome was similar to those set horizontally (65.3 ± 6.4% vs. 59.3 ± 9.2). In a 2 × 2 factorial design trial, Schalkwyk et al. (2000) recorded an increase in hatchability when ostrich eggs were set horizontally for 2 weeks and vertically for the remainder of the incubation period compared to those set vertically for the entire incubation period irrespective of angles of rotation (60° or 90°). Nevertheless, the results of these trials encourage the vertical setting despite having no obvious advantage over the eggs set horizontally then vertically with the appropriate angle of rotation. This is consistent with practices in the industry supported by the work of Funk and Forward (1960) who recorded better hatchability when fowl eggs are set vertically with their air sac up. This position prevents hatching failure due to mal-positioning or pipped eggshells on the narrow end.

### 5.2 Effect on Post-Hatch Performances

**FIGURE 4:**
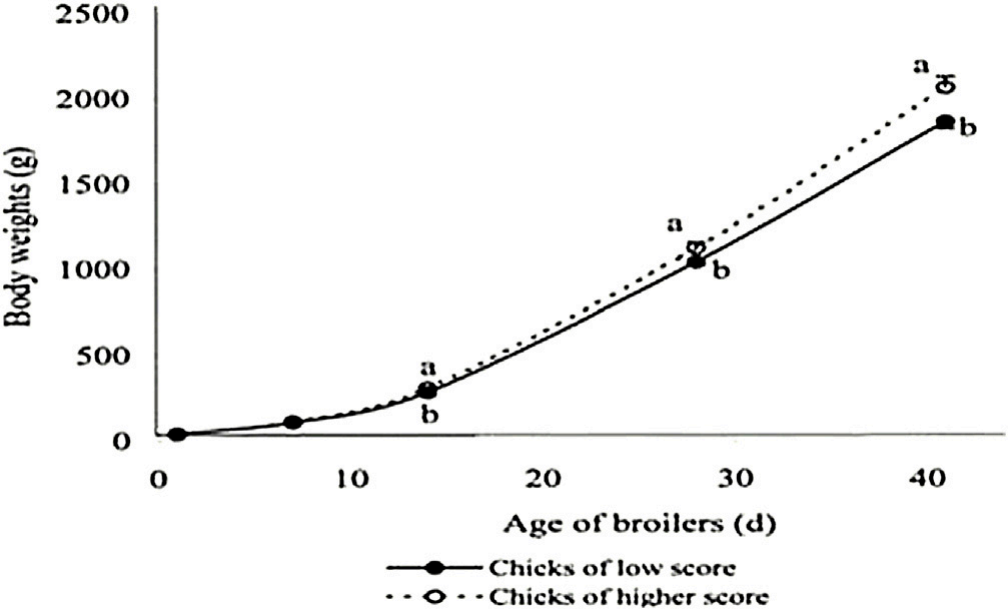
Broiler growth according to day-old chick quality. From Tona et al. (2003a).

Studies on the effect of these different factors on egg turning on post-hatch growth are not well documented. However, many hypotheses can be made based on the results of turning on incubation parameters. Then, Takeshita and McDaniel (1982) observed that although chicks from eggs in horizontal or vertical with small end up required less time to exhibit initial pips, they required longer to emerge from the shell than those in vertical with the small end down. Usually, the spread of hatching can affect the time of first feeding. Shortness of hatching windows when eggs were set with the small end down would lead to improved growth performances through early access to feed, which is crucial for post-hatch performances (Gaglo-Disse et al., 2010; Wang et al., 2014). In addition to short hatching windows, Guo et al. (2021) recorded an increase of goslings of high quality with a proper turning angle. According to Tona et al. (2003a), day-old chick quality and relative growth up to 7 days as well as slaughter performance are positively correlated (Figure 4). The authors concluded that the quality of chicks was better with an adequate turning angle (45°) because these chicks were able to use more nutrients to produce body mass tissue. An adequate turning angle could improve the feed efficiency of birds during the growth period.

## 6 *In ovo* Feeding Practice During the Incubation Process: Effects on Embryo Development and Hatching Performances

In literature, embryos have been fed with a variety of nutrients with a diversity of results depending on the stage of incubation when *in ovo* feeding occurs, the nature and quantity of nutrients inoculated and the route of injection. This paper does not aim to review all the nutrients inoculated in eggs. It focuses on the way *in ovo* feeding of some critical substances that could affect embryo development, physiology and post-hatch growth depending on the different factors mentioned above.

### 6.1 Effect on Physiology, Embryo Development and Hatching Performances

Chicken eggs contain a small amount of carbohydrate (less than 1%) that supplies glucose, the most important source of energy needed for embryo growth (Starck and Rickelefs, 1998). This amount of carbohydrates initially available in the egg could not cover all the needs of the embryo until hatch. Therefore, glucose and glycogen are preferentially utilized as energy sources over lipids and protein because the limited oxygen available is mainly generated *via* gluconeogenesis and glycogenesis (Pearce, 1971). These carbohydrates were important for the final stage of embryonic development especially for pipping and chick emergence from the shell (Christensen et al., 1993; Moran, 2007).

Glucose inoculated in albumen at days 7 during organogenesis failed to improve hatchability while chicks’ weight was significantly improved at hatch (Salmanzadeh et al., 2012). In contrast, *in ovo* injection of carbohydrates (glucose, maltose) during the late stage of incubation (at days 17.5 or days 18) did not affect hatchability or newly chick weight (Ipek et al., 2004; Dos Santos et al., 2010, Eslami et al., 2014). However, Zhai et al. (2011) recorded a decrease in hatchability with glucose, fructose, sucrose or maltose *in ovo* injected at d 18.5 of incubation although body weight or body weight relative to set egg weights were significantly increased. A combination of carbohydrates (maltose, sucrose and dextrin) inoculated *in ovo* on day 19 also increase the bodyweight of newly hatched chicks (Tako et al., 2004). Thus, *in ovo* injection of carbohydrates at the appropriate time during incubation seem to be a good way to improve embryo growth and then chick weight at hatch. In addition, an insufficient amount of glycogen in late-term embryo forces the embryo to mobilize more muscle protein for gluconeogenesis until replenishing of glycogen reserves with the access of newly hatched chicks to feed (Vieira and Moran, 1999a,b; Moran, 2007). Uni et al. (2005) showed that injection of a solution containing β-hydroxy-β-methylbutyrate (a leucine metabolite) into the amniotic fluid of broiler embryos on day 17.5 spare the use of pectoral muscle leads to an increased body-weight at hatch. The *in ovo* inoculation of amino acids mixture seems to be also a good way to improve significantly chick weight at hatch if the amino acids used were identical to the amino acids pattern of egg protein (Al-Murrani, 1982). In chicken embryos, days 19 of embryonic development is an important point when the risk of lipid peroxidation is very high because tissues are characterized by comparatively high levels of polyunsaturated fatty acids. But at this time, the natural antioxidants level is not sufficient for innate protection. This risk is more when internal piping occurs with increasing oxygen availability as pulmonary respiration begins. Thus, low antioxidant status increases the embryo’s susceptibility to lipid peroxidation. However, *in ovo* injection of vitamins (A, B1, B2, B6, and C or E) at days 14 of incubation failed to improve hatchability and chicks’ weight at hatch (Nowaczewski et al., 2012; Goel et al., 2013). In contrast, extract from plants like *Moringa oleifera,* *Nigella sativa, etc*. are rich in carotenoids, an antioxidant naturally present in eggs, increase significantly hatchability (N’nanle et al., 2017; Oke et al., 2021). The advantage of some plant extract use is to concentrate different nutrients like vitamins and trace minerals (selenium, copper, zin, and iron) that act as co-factor of many enzymes involves in hatching success (Malheiros et al., 2012).

### 6.2 Effect on Post-Hatch Performances

Administration of exogenous nutrients and other agents *in ovo* can advance the development of the embryo and post-hatch growth (Uni and Ferket, 2003). Some nutrients *in ovo* injected lead to an increase of body weights at hatch (Al-Murrani, 1982; Tako et al., 2004; Uni et al., 2005; Zhai et al., 2011; N’nanle et al., 2017). Like chick quality, hatching weight is a major predictor of marketing weight in chickens. Thus, advantages observed at hatch in the *in ovo* feeding treatment were maintained during rearing (Al-Murrani, 1982; Uni et al., 2005). This gain could be attributed to changes that occur earlier in the gut. For example, Smirnov et al. (2004) and Tako et al. (2004) showed an increased surface area, length and width of villus at hatch and 3 days after with carbohydrates inoculated at days 17.5 in chicken eggs. In the same way, manna oligosaccharides injected *in ovo* at days 17 resulted in the newly hatched chick with more mature enterocytes in the small intestine that can enhance digestive capacity and epithelial barrier (Cheled-Shoval et al., 2011).

Although chicks’ weight at hatch was unaffected, Joshua et al. (2016) demonstrated that *in ovo* inoculation of nano form of selenium (0.075 or 0.15 µg/egg) and zinc (40 µg/egg) significantly improve weight gain, body weight at market age and feed efficiency (only with nano form of Zn). This could be explained by the involvement of these minerals in immunity (McKenzie et al., 1998; Cardoso et al., 2007). Immunity was also improved by *in ovo* inoculation of probiotics and synbiotics which led to better post-hatch resistance against pathogens (De Oliveira et al., 2014; Sławinska et al., 2014) while prebiotics have been reported to increase the number of beneficial bacteria and promote their early colonization in the intestine of neonatal chicks (Tako et al., 2014).

## 7 Conclusion

There have been remarkable advances in the incubation of chickens. Incubation conditions affect embryo parameters and consequently post-hatch growth differentially according to exposure time and stage of exposure. In addition, the amplitudes of changes in these conditions need to retain attention during incubation. Therefore, classical physical conditions are required to improve hatchability, chick quality and post-hatch growth.
